# Middlesex Hospital

**Published:** 1907-01-19

**Authors:** 


### MIDDLESEX HOSPITAL.

### THE LATE MR. E. A. FARDON.

The death occurred on Wednesday, January 2nd, at St. Lawrence, Ventnor, Isle of Wight, of Mr. E. A. Fardon, who for nearly 30 years had held the appointment of resident medical officer at the Middlesex Hospital. Mr. Fardon came of a Quaker stock, his mother being a Bell of Alton, a descendant of one of the original Quakers, who up to the time of her death, in 1895, at the age of 84 years, regularly wore the Quaker bonnet and grey dress peculiar to that society. She was related by marriage to Elizabeth Fry and the Guernseys of Earlham. One of a family of six, Edward Ashby Fardon was born in 1846, and after receiving his education at the Quaker School at Sidcot, in Somersetshire, entered the salt works owned by his father, Joseph Ashby Fardon, at Droitwich, remaining there until he was 27 years of age, during which time he acquired business habits and knowledge which were of great value to him in after-life. Finding business life uncongenial, he decided to enter the medical profession, and defrayed the cost of his medical training by means of his accumulated savings, subsidised by money earned in the form of scholarships and prizes.

It was thus he came to enter the Middlesex Hospital as a student in 1873, and he qualified M.R.C.S. in 1878, taking the diploma of L.R.C.P. in 1879. He filled the offices of house physician, junior and senior house surgeon, and then of resident obstetric assistant, during his tenure of which the post of resident medical officer became vacant, for which he applied, and which he was selected to fill. Here he found his life-work, and although he had many tempting offers to engage in private practice, for which he was eminently fitted, he refused to avail himself of these, preferring to devote himself with singular whole-heartedness to the interests of the Middlesex Hospital.

Throughout his long term of office he enjoyed the complete confidence of the Board of Management, the staff, resident staff, officials, and nurses, to all of whom he was very willing to render sympathetic assistance. To a great extent at his initiative, and always guided by his counsel and careful supervision, many alterations and reforms were effected in the hospital, the nursing department, and in the school. Amongst these mention may be made of the Resident College for Students, the Trained Nurses' Institute, the Convalescent Home at Clacton-on-Sea, the laundry at Hendon, the removal of the kitchens from the basement to the upper floor of the hospital, additional wards, the operating theatres and sterilising apparatus, the new cancer wing and cancer research laboratories. It may with truth be said that in every department of the hospital there are distinct and lasting evidences of Mr. Fardon's administrative energy and ability. For several years he acted as honorary secretary to the Royal British Nurses' Association, and was of great service in guiding the affairs of that body during a troubled period of its history.

It was close upon two years ago that it was discovered that Mr. Fardon had glycosuria, and in spite of careful dieting and treatment, signs of ageing and of lessened vigour became increasingly apparent. In the summer of last year he contracted a "cold," which proved to be a tuberculous infection of the lungs, and in spite of all that the best advice could suggest and careful attention provide the disease rapidly progressed.

He was laid to rest in the well-known churchyard of the old church of St. Lawrence on Saturday, January 5th, and at the same time a memorial service was held in the chapel of the Middlesex Hospital, which was filled by a large and sorrowing congregation, noticeable amongst whom were his Excellency the Greek Minister, Lord Cheylesmore, and Lord Sandhurst, many members of the weekly Board of Governors of the hospital, the staff, and a large number of past and present students and nurses.

